# Regional Differences in Admission Rates of Emergency Patients Who Visited a Private General Hospital in the Capital City of Cambodia: A Three-Year Observational Study

**DOI:** 10.34172/ijhpm.2021.44

**Published:** 2021-05-19

**Authors:** Yurie Kobashi, Sophathya Cheam, Yoshifumi Hayashi, Masaharu Tsubokura, Veyleang Ly, Chanmakara Noun, Takehiro Kozuma, Buntongyi Nit, Manabu Okawada

**Affiliations:** ^1^Department of General Internal Medicine, Sunrise Japan Hospital Phnom Penh, Phnom Penh, Cambodia.; ^2^Department of Pediatrics, Sunrise Japan Hospital Phnom Penh, Phnom Penh, Cambodia.; ^3^Department of Neurosurgery, Sunrise Japan Hospital Phnom Penh, Phnom Penh, Cambodia.; ^4^Department of Neurosurgery, Kitahara International Hospital, Tokyo, Japan.; ^5^Department of Internal Medicine, Soma Central Hospital, Fukushima, Japan.; ^6^Department of Medicine, University of Puthisastra, Phnom Penh, Cambodia.

**Keywords:** Health Disparity, Regional Disparity, Developing Country, Global Health, Emergency Care

## Abstract

**Background:** Regional disparity is an imperative component of health disparity. In particular, providing emergency care that is equally available in rural areas is an essential part of reducing the urban–rural disparity. The objective of this study was to examine the worsening admission rate among Cambodian emergency patients in a rural area and determine their background characteristics that cause this decline.

**Methods:** To investigate the disparity among patients who visited Sunrise Japan Hospital (SJH), a major general private hospital in the capital, patient data from November 2016 to September 2019 were obtained from the electronic reception patient database. The primary outcome was defined as the proportion of admission patients as an indicator of illness severity. The patients’ addresses were classified into 4 areas based on distance from the capital.

**Results:** A total of 6167 patients who visited the emergency department at SJH between January 2017 and September 2019 were included in the analysis. The proportion of patients who needed to be hospitalized or transferred increased with the distance from the capital. The proportion of patients who finished consultation decreased with the distance from the capital (*P*<.01: Chi-square test). The results of the logistic regression analysis showed that the admission rate in rural areas was significantly higher only among males as compared to that of the capital in multivariate analyses adjusted for age, time, and season.

**Conclusion:** The admission rate of emergency patients who visited a private general hospital in Cambodia’s capital city increased with distance from the capital city. To improve regional disparity among emergency patients, further research is necessary to identify the issues among emergency patients, especially those who are vulnerable.

## Background

**Key Messages**

**Implications for policy makers**

- Gender and age influenced the association between rural–urban residence and hospitalization rates among emergency patients in complex ways. Policy-makers are required to take consideration into vulnerable residents for planning emergency care countermeasure.
- To clarify the health disparity suffered by the socially vulnerable, the system has to be improved to accumulate patient information such as social economic status, disease name, patient level factors, and severity of patients’ diseases at various hospital levels.
- Further investigation of both private and public hospitals is required to determine the health disparities among rural emergency patients, especially for vulnerable residents.

**Implications for the public**

Regional disparity is a major component of health disparity. In particular, providing emergency care that is equally available in rural areas is an essential part of reducing urban–rural disparity. Moreover, the present study found a difference in the admission rates among emergency patients with regards to region, and clarified the patient’s characteristics that are related to regional differences in the admission rates. Thus, it could be implied that healthcare disparity might exist among emergency patients based on regional areas, especially among vulnerable residents.

Health equity is crucial based on ethical considerations and in the prevention of negative social and economic consequences on a national scale.^1^ Health disparity is thought to emerge from the weaknesses of the health systems and individual factors as follows: race, sex, sexual identity, age, disability, socioeconomic status, geographic location, environmental barriers, and stigmatization.^1,2^ In particular, there is a difference between urban and rural health statuses and outcomes, which are usually expressed in terms of utilization, spending, and geographic distribution of providers and services.^3-5^ Thus, regional disparity is an important component of health disparity, and its reduction is a crucial public health issue.

 Several initiatives have been undertaken to reduce the urban–rural disparity until the present time. In particular, providing emergency care, which has a life-saving role for patients with severe disease as a part of primary care, that is equally available in rural areas is an essential part of reducing this disparity.^6,7^ In this context, hospitalization rates varied widely in each area in previous studies in the United States.^8,9^ Moreover, urban locations have been shown to have reduced hospitalization rates.^9^ Furthermore, the gap between urban and rural areas regarding the quality of available treatment is expected to widen in the future, and a comprehensive strategy that addresses the existing issues is needed to overcome this gap.^10^ Even in developing countries, the importance of emergency care is increasing with the shift of the disease burden from communicable diseases to accidents, injuries, and non-communicable diseases.^7,10,11^ Meanwhile, emergency care is being neglected in low- and middle-income countries (LMICs), even though approximately half of the preventable deaths in these countries can be reduced by applying the basic principles of emergency care.^12,13^ In response, global health agencies have foregone the development of emergency systems in favor of primary care to provide care for a larger number of people in LMICs.^14^ Thus, it is vital to identify urban–rural disparities in emergency care as a part of addressing the related existing issues in developing countries. Nevertheless, little research has been conducted in this area.

 Cambodia has been classified as an LMIC by the World Bank. However, over the last 20 years, 16.3 million Cambodian nationals have witnessed considerable improvements in public health, with the country achieving most of the health-related Millennium Development Goals by 2015.^15^ Yet, in 2012, the nation had only 1.4 health workers per 1000 people.^16^ More specifically, some 40% of physicians and 74% of specialist physicians work in the capital city, Phnom Penh,^17^ whereas 80% of the population still reside in communes in rural areas, and 90% of the nation’s poor are rural dwellers.^18^ Besides, private hospitals and clinics, in particular, are concentrated in urban areas; this means that the regional inequity in health services remains a crucial problem,^19^ as illustrated by the fact that the infant mortality rate is approximately three times higher in rural settings than it is in urban settings.^20^ In addition, the emergency department in Cambodia is not recognized as a specialty by the Ministry of Health and does not have a formal training program for specialists in the discipline.^12^ Furthermore, the referral system for emergency patients in Cambodia differs between urban and rural areas. Emergency patients in the capital urban area are mainly transported to public hospitals in the capital, or private hospitals, if patients request it. Meanwhile, emergency patients in rural areas are transported to provincial public hospitals from clinics or health centers first. If patients cannot get adequate treatment in provincial public hospitals, they are transferred to a hospital in the capital. Therefore, Cambodia is an advantageous area to investigate the widening of urban–rural disparities in emergency departments in developing countries with the goal of improving regional disparity.

 The objective of this study was to examine the worsening admission rate among Cambodian rural emergency patients and determine the patient’s background characteristics that affect it. The difference in admission rates in each residential area, as an indicator of medical severity, among emergency patients in private general hospitals in the capital city of Cambodia was investigated by each patient’s background characteristics to clarify regional disparity.

## Methods

 This study used a retrospective observational design.

###  Healthcare Systems in Cambodia

 Approximately 90% of residents in Cambodia are Khmer—the most popular ethnicity. Cambodia has a large area (181 035 km^2^), and transportation time to the capital differs based on the region. Most public hospitals in the capital city have been designated as institution members in 119 emergency systems; however, emergency department classification is not assigned for each private hospital. Moreover, ambulances are widely used as part of emergency patient transport systems.^21^ Nevertheless, many residents use their own cars to transport emergency patients due to the cost and recognition issues of ambulances.^21^ Moreover, Cambodia has both a regulated public health sector and an unregulated private health sector. While a referral system has been developed between provincial hospitals and health centers in public hospitals, there is less of a referral system between private hospitals.^5^ In addition, while public hospitals and health centers are required to be positioned based on the district or number of residents, private hospitals are concentrated in urban areas. Thus, it is essential to investigate patients’ background characteristics and admission rates for each area in general hospitals in the capital city, that both urban and rural patients visit, to understand the regional disparity.

###  Site Detail

 The Sunrise Japan Hospital (SJH) is an international private general hospital in Phnom Penh, the capital city in Cambodia with 14 medical departments, 50 beds for inpatients, an intensive care unit, 2 operating rooms, and endovascular treatment facilities. SJH, which opened in 2016, aims to provide Japanese standard quality medical care in Cambodia. The hospital has 213 multinational personnel, including Cambodian and Japanese staff as well as staff from other countries. More than 90% of the patients are local residents who speak Khmer as their first language, and the patients’ economic statuses mainly range from middle class to affluent. The largest number of foreign patients are Japanese. SJH has a 24-hour emergency department where more than two doctors are always available to see patients. The hospital has advanced facilities, including an operating room with microsurgery equipment, magnetic resonance imaging, intensive care unit, and endovascular treatment room; hence, SJH can accommodate severe status patients. Further, emergency patients can undergo emergency surgery and emergency endovascular treatment if necessary. In addition, SJH engages in social activity in rural areas and continually makes efforts to advertise hospitals in rural areas. For these reasons, SJH is a suitable hospital to identify regional health disparities among emergency patients in Cambodia.

###  Dataset

 To investigate the characteristics of patients who visited SJH, a major general private hospital in Phnom Penh, patient data from November 2016 to September 2019 were obtained from the electronic reception patient database. The patient data included addresses, consultation classification, next plan classification, patients’ primary language, insurance status, visiting date, and visiting time. Information on province was obtained using the author’s manual work from individual patient address records.

###  Data Definition

**Figure 1:**
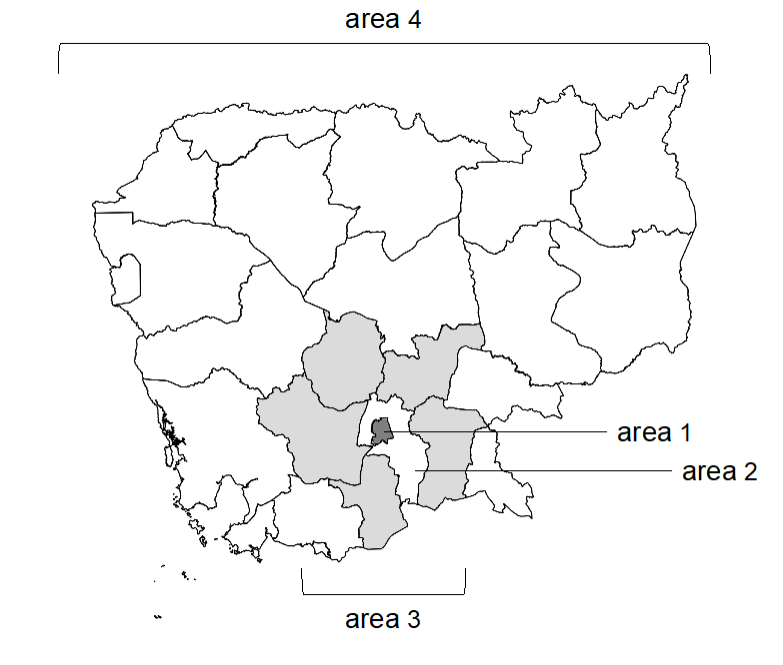
The Four Areas for Analysis Divided by Geographic Location and Distance From the Capital: *Area 1* as Phnom Penh of the capital; *Area 2* as Kandal province, which surrounds the capital city; *Area 3* included five provinces adjacent to Kandal province, (Kampong Chhnang, Kampong Speu, Takeo, Kampong Cham, Prey Veng province); and *Area 4 *included all other provinces.

 Patients of all ages who visited the emergency department between November 2016 and September 2019 were included in this study. The exclusion criteria were foreigners who did not speak the local language as their first language. The residential areas were divided into four areas: Area 1 included Phnom Penh, which is the capital; Area 2 included Kandal province, which surrounds the capital city; Area 3 includes five provinces adjacent to Kandal province (Kampong Chhnang, Kampong Speu, Takeo, Kampong Cham, Prey Veng province); and Area 4 included all other provinces (Figure 1). The distance from Phnom Penh is greater in the ascending order for Areas 2, 3, and 4. It is also worth noting that in the country, a year is divided into two seasons: the rainy season from May to October and the dry season from November to April.

###  Primary Outcome

 The primary outcome was defined as the proportion of admission patients as an indicator of illness severity. Transfers were included as part of the admission for statistical analysis because most patients who needed transfers had severe statuses and required hospitalization after transfer.^22^ The admission rate was set as the primary outcome because the admission rate of emergency patients has been surveyed in many countries and regions. This means that the admission rate might be a valid indicator of the severity of illness and that the results’ comparison with other regions will be easy. Moreover, in our literature review, information on the admission rate of emergency patients in private hospitals in the capital of Cambodia was not found. Thus, the primary outcome was investigated using patient background characteristics such as gender and age groups.

###  Analysis

 First, the patients’ background characteristics were analyzed descriptively, and chi-square test or Mann-Whitney U test were performed to compare the results based on gender. Next, the shift in the number of emergency patients in each area was determined. Then, the proportions of admission rate and consultation completion rate (next plan classification), gender, visiting time, and season for each of the four areas were descriptively analyzed. A chi-square test was performed for each classification based on address area. In addition, a logistic regression analysis was performed for univariate and multivariate analyses. A univariate analysis was performed to determine whether age, sex, address area, time, and season affect the admission rate for emergency patients. The multivariate logistic regression model was constructed with age, gender, address area, time, and season as independent variables and the admission rate as the dependent variable. The effect of address area on admission rate after adjusting the age, gender, time, and season variables was determined. Further, the effect of address area on admission rate after adjusting the age, gender, time, and season variables was determined by each gender group and age group with the same multivariate logistic regression model. To compensate for missing information in terms of address area, another multivariate logistic regression model—which added the missing values to the area variable as one of the components—was constructed. The results using the multivariate logistic models with and without missing values were almost the same (Table S1, Supplementary file 1). Adjusted R2, goodness of fit of the models, was 0.104 for the model with missing values and 0.134 for the model without missing values (Table S1). Based on these results, it was assumed that the deficiencies occurred randomly and that the presence of bias associated with deficiencies was small.

 A statistically significant *P* value was set at.05. STATA IC (Lightstone, Texas, USA, version 15) was used for the analysis. All methods were carried out in accordance with the STROBE guidelines.

## Results

 A total of 6167 patients who visited the emergency department at SJH between January 2017 and September 2019 were included in the analysis. Patient characteristics are shown in Table 1. There was a higher percentage of males (51.97%) in the group. The median age (25th, 75th centiles) was 59 [36, 73]. The most common next plan classification was completion of consultations (44.97%). Only 0.62% of the participants used insurance.

**Table 1:** Participant’s Characteristics (N = 6167)

	**No. (%)**
Gender	
Male	3205 (51.97)
Female	2962 (48.03)
Age (Median, [interquartile range])	59 [36–73]
Address	
Area 1 (Phnom Penh)	1428 (23.16)
Area 2 (Kandal)	147 (2.38)
Area 3 (Close to Kandal)	333 (5.40)
Area 4 (Other province)	320 (5.19)
Unknown	3939 (63.87)
Insurance	
Yes	38 (0.62)
No	6129 (99.38)
Next plan classification	
Completed consultation	2773 (44.97)
Revisit for medicine	81 (1.31)
Revisit for consultation	1078 (17.48)
Admission	1157 (18.76)
Transfer	67 (1.09)
Unknown	1011 (16.39)
Time	
0:00-7:59	924 (14.98)
8:00-11:50	883 (14.32)
12:00-17:59	1709 (27.71)
18:00-23:59	2651 (42.99)
Season	
Rainy season	3495 (56.67)
Dry season	2672 (43.33)
Out of Pocket Payment (Median, [interquartile range])	217.76 [100.62–358.06]

 Patients characteristics by gender groups are summarized in Table S2. The proportion of patients who needed to be hospitalized or transferred was higher in male than female groups (*P* < .01: chi-square test). Out of pocket payment was also higher in males than females (*P* < .01: Mann-Whitney U test).

**Figure 2:**
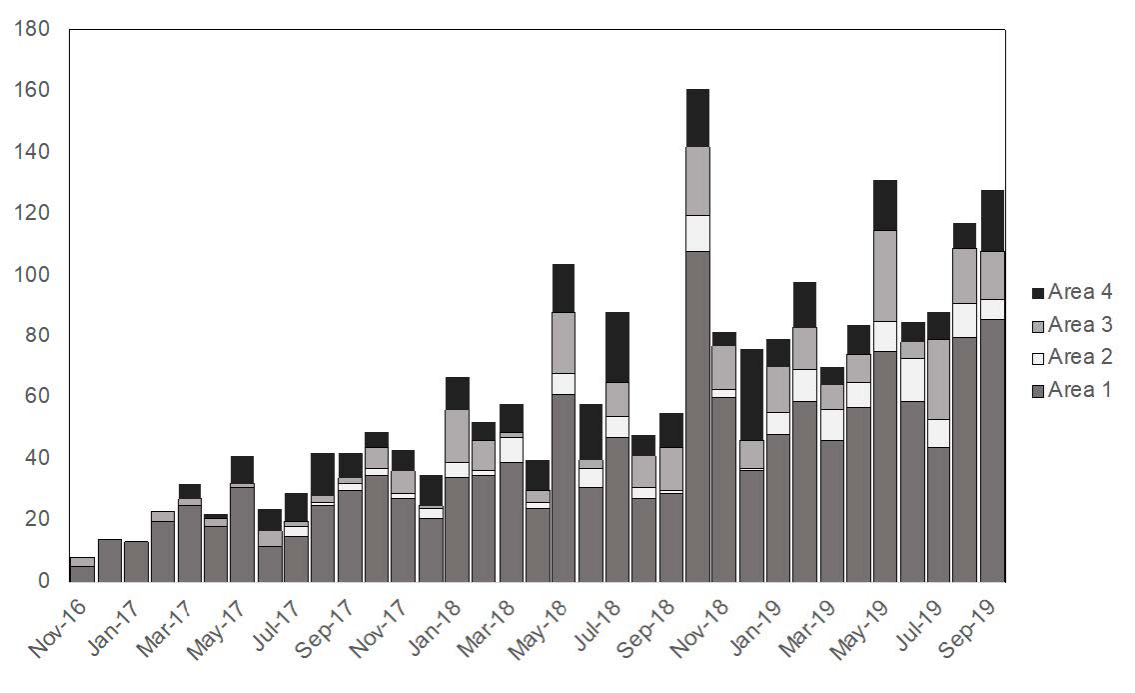
The Number of Emergency Department Patients in Each Geographic Area Group Every Month.

Figure 2 shows the monthly number of emergency patients based on the address area. The number of patients increased over the three years. The proportion of patients coming from Areas to 2–4 increased gradually during the first eight months; however, the proportion became constant after that period. The proportion of patients coming from outside of Phnom Penh was 94.92% from November to December 2016, 75.00% from January to June in 2017, and was maintained at approximately 55% after that period.

Table 2 shows the proportion of patients based on area, including admission rate of emergency patients, gender, age, visiting time, and season. The proportion of patients who needed to be hospitalized or transferred was increased with the distance from Phnom Penh. Meanwhile, the proportion of patients who finished their consultations decreased with the distance from Phnom Penh (*P* < .01: chi-square test). The most common visiting time for patients living in Area 1 was 18:00–23:59, whereas the most common visiting time for patients living in Area 4 was 12:00–17:59.

**Table 2:** Proportion of Admission Rate of Emergency Patients by Gender, Age, Time, Season, and Address Area

	**Area 1 **	**Area 2**	**Area 3**	**Area 4**
Gender				
Male	776 (54.34)	82 (55.78)	170 (51.05)	169 (52.81)
Female	652 (45.66)	65 (44.22)	163 (48.95)	151 (47.19)
Age (Median, [interquartile range])	56 [33–73]	66 [52–73]	65 [46–73]	61 [52–74]
Next classification^a^				
Consultation completion	695 (56.18)	71 (55.47)	115 (40.93)	106 (40.15)
Revisit	282 (22.80)	26 (20.31)	71 (25.27)	59 (22.35)
Admission, transfer	260 (21.02)	31 (24.22)	95 (33.81)	99(37.50)
Time^a^				
0:00-7:59	222 (15.55)	16 (10.88)	46 (13.81)	47 (14.69)
8:00-11:50	168 (11.76)	24 (16.33)	62 (18.62)	39 (12.19)
12:00-17:59	396 (27.73)	36 (24.49)	98 (29.43)	118 (36.88)
18:00-23:59	642 (44.96)	71 (48.30)	127 (38.14)	116 (36.25)
Season				
Rainy season	807 (56.51)	87 (59.18)	189 (56.76)	204 (63.75)
Dry season	621 (43.49)	60 (40.82)	144 (43.24)	116 (36.25)
Out of Pocket Payment (Median, [interquartile range])	209.65 [103.47-103.47]	276.53 [129.00-416.29]	287.95 [157.10-392.69]	309.80 [178.66-430.71]

^a^ *P* <.01 with Chi-square test.

 The result of the univariate and the multivariate analyses for whole group is shown in Table S3. The results of the multivariate analyses adjusted for gender, age, time, and season showed that the admission rates of address areas 3 and 4 was significantly increased as compared to the admission rates of address area 1.

Table 3 shows the results of the logistic regression analysis by gender to determine whether the address area affects the admission rate. The results of multivariate analyses adjusted for age, time, and season showed that the admission rates of address areas 3 and 4 was significantly increased as compared to the admission rates of address area 1 only among males.

**Table 3:** Multivariate Logistic Regression Analysis by Gender to Determine Whether the Address Area Affects the Admission Rate

	**Multivariate Analysis, Adjusted OR (95% CI)**	
	**Male (n = 1.001)**	**Female (n = 909)**
Age	1.03 (1.02-1.04)^a^	1.04 (1.03-1.05)^a^
Address		
Phnom Penh	Ref.	Ref.
Kandal	1.21 (0.70-2.09)	0.58 (0.26-1.30)
Close to Kandal	2.62 (1.72-3.99)^a^	1.11 (0.72-1.72)
Other	3.57 (2.36-5.40)^a^	1.06 (0.66-1.71)
Time		
8:00-11:50	Ref.	Ref.
12:00-17:59	2.83 (1.50-5.33)^a^	1.56 (0.82-2.95)
18:00-23:59	3.68 (1.96-6.91)^a^	2.60 (1.44-4.70)^a^
0:00-7:59	3.43 (1.73-6.81)^a^	3.18 (1.62-6.26)^a^
Season		
Rainy season	Ref.	Ref.
Dry season	1.01 (0.75-1.38)	0.84 (0.59-1.19)

Abbreviation: OR, odds ratio. ^a^ *P* <.01.

Table 4 shows the results of the logistic regression analysis to determine whether the address area affects the admission rate by age group. The results of multivariate analyses adjusted for gender, time, and season showed that the admission rates of address areas 2, 3 and 4 was significantly increased as compared to the admission rates of address area 1; the result was positively significant among the 20–59-year-old age group, whereas it was negatively significant among the over-59-years-old age group.

**Table 4:** Multivariate Logistic Regression Analysis by Age Groups to Determine Whether the Address Area Affects to the Admission Rate

	**Multivariate Analysis, Adjusted OR (95% CI)**	
	**20-59 Years Old ** **(n = 791)**	**Over 59 Years Old ** **(n = 962)**
Gender		
Male	Ref.	Ref.
Female	0.57 (0.38-0.85)^a^	0.58 (0.44-0.76)^a^
Address		
Phnom Penh	Ref.	Ref.
Kandal	2.40 (1.16-4.95)^b^	0.55 (0.31-0.98)^b^
Close to Kandal	2.05 (1.13-3.71)^b^	1.55 (1.08-2.21)^b^
Other	3.20 (1.95-5.25)^a^	1.64 (1.12-2.40)^b^
Time		
8:00-11:50	Ref.	Ref.
12:00-17:59	1.95 (0.92-4.14)	2.81 (1.58-4.99)^a^
18:00-23:59	2.54 (1.23-5.25)^b^	4.21 (2.40-7.38)^a^
0:00-7:59	2.37 (1.04-5.42)^b^	3.34 (1.80-6.20)^a^
Season		
Rainy season	Ref.	Ref.
Dry season	0.59 (0.39-0.90)^b^	0.91 (0.68-1.21)

Abbreviation: OR, odds ratio. ^a^ *P* <.01, ^b^*P* <.05,

## Discussion

 It is a crucial public health issue to identify urban and rural disparities in the emergency department to reduce healthcare disparities. This study aimed to determine whether the admission rate is worsening among Cambodian rural emergency patients and determine patients’ background characteristics that affect it.

 Moreover, gender and age influenced the association between rural-urban residence and hospitalization rates in complex ways. The association between address of residence and hospitalization rate was greater among males and younger patients. Considering that there was no significant difference in the number of patients by gender groups, female patients from rural areas might have minor illnesses that did not require hospitalization; or female patients from rural areas might have chosen outpatient treatment or consultation because they had financial or other problems. The same assumption could be made regarding age groups as well. To clarify the health disparities suffered by the socially vulnerable, improving the system to accumulate patient information such as economic status and disease name is required at various hospital levels.

 It was found that the proportion of the admission rate, which might be associated with disease severity, increased with distance from the capital. The logistic regression analysis results for all participants showed that the hospitalization rate in the farthest address area from the capital was 2.1 times that of the capital. The fact that out-of-pocket payments increased with the distance from the capital did not conflict with the results. A study on the admission rate of emergency patients receiving Medicare across the United States in 2016 showed that the admission rate varied widely based on region, sometimes even doubling; however, the cause of this disparity has not been clarified.^8^ Besides, the previous study showed that racial differences affected the admission rates for heart failure,^23^ and that being young males from deprived areas affected emergency hospital admission rate for violence.^24^ Based on the fact that the present study showed a difference in admission rate by region as well, and the fact that the emergency transport systems of the rural areas are fragile,^21^ it can be inferred that the differences between regions might affect the health statuses of emergency patients in rural areas. Further research is thus required to determine the health disparities of emergency patients. This includes gathering information on the diseases, ailments, conditions, and other specific details of individual cases.

 Moreover, it was found that the number of emergency patients visiting SJH has increased in the three-year period being studied in both urban and rural areas. In Cambodia, medical tourism in which patients visit neighboring countries such as Thailand,^25^ Vietnam, and Singapore for more advanced medical care is becoming a trend. However, some patients changed their minds and came to SJH instead of going to hospitals abroad (data not shown). Moreover, due to the COVID-19 pandemic, various barriers have appeared to going to hospitals abroad; consequently, a system through which patients can be transported for medical care to domestic hospitals might be required, especially for emergency departments and departments that have minimal time for life-saving. Until the present time, the Ministry of Health in Cambodia has taken measures to improve the patient transportation system and raise awareness on emergency calls in the community. Four elements are required to improve the emergency system in Cambodia as per previous reports: access to transportation methods, triage, inter-institution cooperation and communication, and pre-hospital care.^21^ Further, the emergency department can be a potential target department for increasing the proportion of patients who receive medical care in domestic hospitals.

 The admission rate of patients who visited SJH was lower than that of provincial public hospitals in rural areas. The admission rate of patients who visited SJH was about 20%, whereas the admission rate of patients who visited provincial public hospitals (Battambang Provincial Hospital, Sampov Meas Provincial Hospital) in rural areas was 60%.^12^ The proportion of admission rate at 60% was higher as compared to the admission rate in developed countries.^8,22^ This difference might be due to the following reasons: the SJH has substantial facilities and equipment for diagnosis, which reduces the proportion of hospitalized patients; medical costs in private hospitals are expensive compared to public hospitals; and the severity of patients’ conditions was mild as compared to that in public hospitals. Nevertheless, further investigation on the causes of this difference is required through the examination of both private and public hospitals.

 When interpreting these findings, several limitations should be considered. First, consultation bias should be considered because the present research is a single-facility study in a private hospital. Second, many address data were not identified. Since entering address information was left to the individual patient, there was a large amount of incomplete information with only street address data and/or erroneous data. Third, obtaining the information on social status, underlying causes of the disparities, patient level factors, and severity of patients’ diseases was insufficient. Previous studies have shown that wealth inequality and inequality in healthcare access caused health disparities, especially in developing countries.^26^ On the other hand, the use of electronic medical records and electric databases is inadequate in Cambodia, and the standards for recording primary patient information such as disease names are vague; therefore, this information could not be sufficiently obtained. A cohort study to investigate how these underlying causes of the disparities affect the hospitalization rates might be the most crucial further research. Fourth, the present study could not obtain the data on length of stay, health outcomes, complications, and mortality for comparison with the resident addresses. Further study is required to examine the likelihood of admission of rural people based on this information. Fifth, one of the most crucial limitations was that this study did not include information on causes of admissions. Despite these limitations, the present study is the first to provide information on the correlation between the address area and admission rate of emergency patients in a private general hospital in the capital to improve the regional disparity among emergency patients in Cambodia.

## Conclusion

 The admission rate of emergency patients who visited a private general hospital in Cambodia’s capital city increased with distance from the capital city, especially among males. To improve regional disparity among emergency patients, further research is required to identify the issues among rural emergency patients especially those who are vulnerable.

## Acknowledgement

 We would like to thank Mr. Kamata Keiya and Mr. Takahiro Yoshimi and all staff members at the SJH, Phnom Penh for providing critical data for this research.

## Ethical issues

 All experimental protocols in the present study were approved by the National Ethics Committee for Health Research in Cambodia (Ethics Committee, ID: 079NECHR). Individual informed consent was waived by the National Ethics Committee for Health Research in Cambodia, because the opt-out consent process was used in this study.

## Competing interests

 Authors declare that they have no competing interests.

## Authors’ contributions

 All the authors made a substantial contribution to this research. YK, MT, and MO contributed to the writing the paper, while all members contributed to the study design, data collection, and coordination with local stakeholders.

## Funding

 This research did not receive any specific grant from any funding agency in the public, commercial, or non-profit sector.

## Supplementary files

Supplementary file 1 contains Tables S1-S3.
