# Supplementary material for: Regional Differences in Admission Rates of Emergency Patients Who Visited a Private General Hospital in the Capital City of Cambodia: A Three-Year Observational Study
Source: Int J Health Policy Manag. 2021 May 19;11(8):1425–31. doi: 10.34172/ijhpm.2021.44 (PMC9808335; doi:10.34172/ijhpm.2021.44)
Supplement: Supplementary file 1 — contains Tables S1-S3. [file ijhpm-11-1425-s001.pdf]

Article title: Regional Differences in Admission Rates of Emergency Patients Who Visited a Private General Hospital in the Capital City of Cambodia: A Three-Year Observational Study

Journal name: International Journal of Health Policy and Management (IJHPM)

Authors' information: Yurie Kobashi[1]*, Sophathya Cheam[2], Yoshifumi Hayashi[3,4], Masaharu Tsubokura[5], Veyleang Ly[1], Chanmakara Noun[1], Takehiro Kozuma[1], Buntongyi Nit[6], Manabu Okawada[2]

[1]Department of General Internal Medicine, Sunrise Japan Hospital Phnom Penh, Phnom Penh, Cambodia.

[2]Department of Pediatrics, Sunrise Japan Hospital Phnom Penh, Phnom Penh, Cambodia.

[3]Department of Neurosurgery, Sunrise Japan Hospital Phnom Penh, Phnom Penh, Cambodia.

[4]Department of Neurosurgery, Kitahara International Hospital, Tokyo, Japan.

[5]Department of Internal Medicine, Soma Central Hospital, Fukushima, Japan.

[6]Department of Medicine, University of Puthisastra, Phnom Penh, Cambodia.

(*Corresponding author: yurie-s@umin.ac.jp)

Supplementary file 1

Table S1. Multivariate logistic regression analysis with missing values of address.

	Multivariate analysis (N = 5,155)<br>Adjusted OR (95% CI)
**Gender**	
Male	Ref.
Female	0.70 (0.61–0.80) **
**Age**	1.03(1.03–1.03) **
**Address**	
Phnom Penh	Ref.
Kandal	0.94 (0.60–1.47)
Close to Kandal	1.80 (1.33–2.42) **
Other	2.02 (1.50–2.73) **
Unknown	1.15 (0.97–1.36)
**Time**	
8:00–11:50	Ref.
12:00–17:59	3.29 (2.52–4.29) **
18:00–23:59	3.22 (2.49–4.17) **
0:00–7:59	2.51 (1.87–3.37) **
**Season**	
Rainy season	Ref.
Dry season	0.91 (0.79–1.05)

* $P < 0.05$, ** $P < 0.01$

OR = Odds ratio

CI = Confidence interval

Table S2. Patients characteristics by gender groups. (N = 6,167)

	Male (n = 3,205)	Female (n = 2,962)
**Age** (Median, [interquartile range])	58 [37–73]	60 [36–73]
**Insurance**		
Yes	20 (0.62)	18 (0.61)
No	3185 (99.38)	2944 (99.39)
**Next plan classification** (n = 5,156) **		
Consultation finish	1423 (53.64)	1431 (57.17)
Revisit	535 (20.17)	543 (21.69)
Admission, transfer	695 (26.20)	529 (21.13)
**Time**		
0:00–7:59	498 (15.54)	426 (14.38)
8:00–11:50	457 (14.26)	426 (14.38)
12:00–17:59	879 (27.43)	830 (28.02)
18:00–23:59	1371 (42.78)	1280 (43.21)
**Season**		
Rainy season	1823 (56.88)	1672 (56.45)
Dry season	1382 (43.12)	1290 (43.55)
**Out of Pocket Payment** (Median, [interquartile range]) **	230.80 [101.82–374.47]	207.23 [100.41–339.20]

* $P < 0.05$, ** $P < 0.01$ with Chi-squire test or Mann-Whitney U test

Table S3. Multivariate logistic regression analysis to determine whether the address area affects to the admission rate.

	Univariate analysis<br>Unadjusted OR (95% CI)	Multivariate analysis (N=1,910)<br>Unadjusted OR (95% CI)
**Gender**		
Male	Ref.	Ref.
Female	0.75 (0.66–0.86) **	0.60 (0.50-0.75) **
**Age**	1.03 (1,03–1.03) **	1.03(1.03–1.04) **
**Address**		
Area 1	Ref.	Ref.
Area 2	1.20 (0.78–1.84)	0.92 (0.59–1.44)
Area 3	1.92 (1.45–2.55) **	1.81 (1.34–2.44) **
Area 4	2.25 (1.70–3.00) **	2.10 (1.55–2.84) **
**Time**		
8:00–11:50	Ref.	Ref.
12:00–17:59	3.55 (2.74–4.60) **	2.18 (1.40–3.39) **
18:00–23:59	3.07(2.39–3.95) **	3.08 (2.01–4.72) **
0:00–7:59	2.45(1.84–3.27) **	3.20 (1.99–5.16) **
**Season**		
Rainy season	Ref.	Ref.
Dry season	0.89 (0.78–1.01)	0.91 (0.72–1.14)

* P < 0.05, ** P < 0.01

OR = Odds ratio

CI = Confidence interval
